# Treatment of, and *Candida utilis* biomass production from *shochu* wastewater; the effects of maintaining a low pH on DOC removal and feeding cultivation on biomass production

**DOI:** 10.1186/2193-1801-2-514

**Published:** 2013-10-06

**Authors:** Takashi Watanabe, Haruyuki Iefuji, Hiroko K Kitamoto

**Affiliations:** National Institute for Agro-Environmental Sciences (NIAES), 3-1-3 Kannondai, Tsukuba, Ibaraki, 305-8604 Japan; Research Fellow of the Japan Society for the Promotion of Science, 1-8 Chiyoda-ku, Tokyo, 102-8472 Japan; National Research Institute of Brewing (NRIB), 3-7-1 Kagamiyama, Higashihiroshima, Hiroshima, 739-0046 Japan; Ehime University, 10-13 Dogo-himata, Matsuyama, Ehime, 790-8577 Japan

**Keywords:** *Candida utilis*, *Shochu* wastewater, Treatment, Biomass production, Amino acid composition

## Abstract

*Shochu* wastewater (SW; alcoholic distillery wastewater) contains large amounts of organic compounds (25,000 – 60,000 COD mg/L), nitrogen (1,000 – 6,000 T-N mg/L), and phosphorus (500 – 1,000 T-P mg/L). Despite its high nutrient content, SW is highly perishable, which limits its utilization for animal feed and fertilizer. Therefore, SW is mainly treated by methane fermentation. On the other hand, a feed yeast, *Candida utilis*, can utilize various organic compounds and be utilized as a yeast extract source and animal feed. We previously bred a mutant, *C. utilis* UNA1, that accumulates a large amount of nitrogen. Here, we investigated the use of *C. utilis* UNA1 to treat highly concentrated SW. With fed-batch cultivation using a 5-L jar fermenter, controlling pH at 5.0 with H_2_SO_4_, 62.9% of DOC, 38.4% of DTN, and 44.5% of DTP were stably removed from non-diluted barley *shochu* wastewater (BSW), and about 16.7 kg of freeze-dried yeast biomass was obtained. The yeast sludge biomass generated from BSW contains about 60% crude protein. Furthermore, using H_2_SO_4_ to control pH increased the sulfur content of wastewater, which increased the methionine composition of yeast sludge biomass.

## Introduction

*Shochu* is a traditional Japanese distilled liquor made from barley, sweet-potato, rice, and other crops. In the south Kyushu region, the annual discharge of *shochu* waste is about 689,000 ton/year. Although *shochu* waste contains large amounts of nutrients (Ikeda et al. 2012), its high perishability limits its use for animal feed and fertilizer (Tsuyumu et al. 2003). *Shochu* waste has high concentrations of suspended solids (30,000 – 65,000 SS mg/L), organic compounds (25,000 - 60,000 COD mg/L), nitrogen (1,000 – 6,000 T-N mg/L), and phosphorus (500 – 1,000 T-P mg/L). This makes it too expensive to treat by the conventional activated sludge method. The main treatment method of *shochu* waste is solid–liquid separation, and the liquid part (*shochu* wastewater; SW) is treated by combining a methane fermentation process, a physical phosphorus removing process, and a conventional activated sludge process.

Previously, the National Research Institute of Brewing (NRIB) of Japan developed an aerobic wastewater treatment method using a combination of yeasts and activated sludge (Yoshizawa 1978). This system removes large amounts of organic compounds, requires little space, and discharges little waste sludge. This method is useful for treating food and beverage industry wastewater (Yoshizawa 1978) and has also been used to treat SW in laboratory experiments for over thirty years (Saito et al. 1983; Suzuki et al. 1991; Watanabe et al. 2009; Yoshii et al. 2001).

The wastewater treatment abilities of yeast strains can be improved by non-recombinant techniques. We found that phenotypes of the *PHO* regulatory system of *Saccharomyces cerevisiae* (Oshima 1997) are useful for improving the phosphorus accumulation capacity (Watanabe et al. 2008). We also found that positive selection of methylamine-resistant mutants to isolate *URE2* mutants (Kamerud & Roon 1986) was useful for finding strains with improved nitrogen accumulation ability (Watanabe et al. 2013b). We also confirmed this method can apply to a wastewater treatment yeast *Hansenula anomala* (Watanabe et al. 2013c).

However, it is necessary to keep these yeasts as the dominant microorganism in the yeast treatment tank by adding chemicals, such as HClO and HCl (Yoshizawa et al. 1980), and by replacing the yeast sludge with fresh seed yeast sludge at regular intervals (Watanabe et al. 2009). On the other hand, treating wastewater with yeast is also considered an attractive way to produce yeast biomass resource. For example, cheese whey was used to produce a food yeast, *Kluyveromyces fragilis* (Paul et al. 2002). Yeast biomass productivities of *Debaryomyces hansenii*, *Kluyveromyces marxianus*, and *Pichia stipitis* were investigated using brewery’s spent grains hemicellulosic hydrolyzates (Duartc et al. 2008). A feed yeast *Candida utilis* was used to produce biomass from salad oil manufacturing wastewater (Zheng et al. 2005). Furthermore, the effect of *C. utilis* on the degradation of forages was investigated (Ando et al. 2006).

However, these studies did not investigate the stability of continuous cultivation and the wastewater treatment abilities. Because wastewater is discharged continuously over long periods, the treatment method must be highly stable. In order to continuously treat SW and produce yeast biomass at the same time, we previously bred a mutant *C. utilis* UNA1 with high nitrogen-accumulating ability (Watanabe et al. 2013a). In that study, we also demonstrated repeated-batch cultivation using barley *shochu* wastewater (BSW) (two times diluted) and obtained about 10 g dry yeast sludge biomass per liter of wastewater. Because dilution of SW requires large scale reactors and high operational costs, further works to efficiently treat undiluted SW are needed for practical application.

Here, we investigated the use of *C. utilis* UNA1 to treat undiluted SW. We found that we could do this by controlling the pH and continuously feeding into the reactor. We also estimated the components of yeast sludge biomass.

## Materials and methods

### Strains and culture conditions

*Candida utilis* IFO1086 strain and its highly-nitrogen-accumulating mutant UNA1 (Watanabe et al. 2013a) were used in this study. These strains were maintained on YM agar plates (0.3% yeast extract, 0.3% malt extract, 0.5% peptone, and 1% glucose, and 2% agar) at 4°C.

YM medium (0.3% yeast extract, 0.3% malt extract, 0.5% peptone, and 1% glucose) was used for pre-cultivation. Barley *shochu* and sweet potato *shochu* waste were centrifuged at 7,000 rpm for 10 min to obtain the supernatants. These *shochu* wastes were discharged from pot distillers. Each supernatant was used as SW for jar fermenter cultivation experiments. The physico-chemical characteristics of each SW used in this study are shown in Table 1.

**Table 1:** **Physico-chemical characteristics of** ***shochu*** **wastewater used in this study**

	Barley	Sweet-potato
pH (−)	3.96	4.05
COD_Mn_ (mg/l)	47,000	17,400
DOC (mg/l)	42,000	16,000
Total sugar (mg/l)	1,700	3,500
Glycerol (mg/l)	2,200	1,500
Organic acids (mg/l)	26,400	3,500
DTN (mg/l)	6,100	1,100
DTP (mg/l)	800	290
K (mg/l)	420	1,800
Ca (mg/l)	100	300
Mg (mg/l)	100	120

### Analytical methods

The absorbance at 660 nm (OD_660_) was measured in order to determine cell density using a spectrometer (Ultrospec 2000, Pharmacia Biotech Co. Ltd., Cambridge, England). Dissolved organic carbon (DOC) was analyzed using a TOC analyzer (TOC-5000A, Shimazu Co. Ltd., Kyoto, Japan). Nitrate (NO_3_-N), Dissolved total nitrogen (DTN), inorganic phosphate (PO_4_-P), and total phosphorus were analyzed according standard methods (APHA, AWWA, WEF 1998). pH was measured using a pH meter (HM-30S, TOA DKK Co. Ltd., Tokyo, Japan).

### Repeated-batch cultivation using a 1.5-L jar fermenter

The yeast strains were pre-incubated in a test tube containing 5 mL of YM medium with shaking at 30°C for 24 h. The pre-culture was inoculated into 1 L of two-times-diluted sweet-potato *shochu* wastewater (SSW) in a 1.5-L jar fermenter. The cultivation conditions were agitating at 400 rpm, aerating at 0.7 LPM, and incubating at 30°C. After 24 h cultivation, 0.5 liters of culture was replaced with new 0.5 liters of SSW every 12 h. The replacing cycles were repeated 30 times for *C. utilis* UNA1 and 12 times for *C. utilis* IFO1086.

Thus, thy hydraulic retention time (HRT) and the sludge retention time (SRT) were 24 h. The DOC-volume loading of SSW was about 16 kg/m^3^/d.

### pH controlled-batch cultivation using a 5-L jar fermenter

*C. utilis* UNA1 was pre-incubated in 50 mL YM medium using a 300-mL flask with shaking at 120 rpm, at 30°C for 24 h. The pre-culture was inoculated into 3 L of 5-times-diluted BSW. The cultivation conditions were agitating at 400 rpm, aerating at 0.5 LPM, and incubating at 30°C. When pH exceeded 5.0, H_2_SO_4_ solution (0.49%) was automatically added to maintain pH value at 5.0.

### Repeated-fed-batch cultivation using a 5-L jar fermenter

*C. utilis* UNA1 was pre-incubated in 50 mL YM medium using a 300-mL flask with shaking at 120 rpm, at 30°C for 24 h. The pre-culture was inoculated into 2 L of 5-times-diluted BSW. The cultivation conditions were agitating at 400 rpm, aerating at 0.5 LPM, incubating at 30°C, and pH value was maintained at 5.0 using H_2_SO_4_ solution (0.49%). After 24 h cultivation, BSW was continuously feeding at 2 L/24 h ratio, and 2 L of culture was removed at every 24 h. After 4 times repeated this cycle, the feeding wastewater was changed to 1:1 mixture of BSW and SSW (mixed *shochu* wastewater; MSW). Feeding ratio and removing time were also changed to 2 L/20 h and 20 h, respectively. This cycle was also repeated 4 times.

Thus, the hydraulic retention time (HRT) and the sludge retention time (SRT) of BSW and MSW 48 h and 40 h, respectively. The DOC-volume loadings of BSW and MSW were 20.9 kg/m^3^/d and 18.9 kg/m^3^/d, respectively.

### Component analysis of yeast sludge biomass

Culture from the jar fermenter cultivation was centrifuged at 7,000 rpm for 10 min to obtain yeast sludge biomass. The yeast sludge biomass was freeze-dried using a BenchTop 4 K Freeze dryers (model 4KBTZL, SP Industries, NY, USA). The freeze-dried yeast sludge biomass was analyzed for protein, fat, fiber, soluble non-nitrogen, and ash according to the manual of Association of Official Analytical Chemists (AOAC 1980). Amino acid analysis was performed by hydrolyzing the freeze-dried yeast sludge biomass with 6 N HCl at 110°C for 22 h and analyzing the hydrolysate with an amino acid auto-analyzer.

## Results and discussion

### Repeated-batch cultivation of sweet-potato *shochu* wastewater

BSW contains more organics and total nitrogen than other kinds of SW. Its discharge volume per year is also the largest. We previously confirmed that *C. utilis* UNA1 could efficiently and stably treat BSW by repeated-batch cultivation using a 1.5-L jar fermenter (Watanabe et al. 2013a). The discharge volume per year of SSW is almost the same as that of BSW. We first investigated the efficiency of SSW using a 1.5-L jar fermenter.

With 30-cycles repeated-batch cultivation, *C. utilis* UNA1 stably utilized and removed DOC, DTN and DTP from SSW (Table 2). The amounts of DOC, DTN, and DTP removed by *C. utilis* UNA1 were higher than those removed by *C. utilis* IFO1086 (Table 2). These results were similar to previous results using BSW (Table 2, (Watanabe et al. 2013a)), indicating that organic compounds of SSW are also good substrates for *C. utilis* biomass production. On the other hand, relatively low DOC of SSW causes relatively low biomass (OD_660_) production (Table 2). Thus, SSW would have to be concentrated or mixed with thick substrate to increase biomass productivity.

**Table 2:** **Summary performances of repeated-batch cultivation by** ***C. utilis*** **UNA1 and** ***C. utilis*** **IFO1086**

		Sweet-potato ***shochu*** wastewater		Barley ***shochu*** wastewater^a^	
		UNA1	IFO1086	UNA1	IFO1086
Periods	Cycles	30	12	50	12
DOC	Influent (mg/l)	15877 ± 740	15621 ± 1201	19731 ± 311	18444 ± 557
	Effluent (mg/l)	7691 ± 545	8179 ± 927	8075 ± 314	8217 ± 495
	Removal ratio (%)	51.6 ± 3.4	47.6 ± 5.9	59.1 ± 1.6	55.5 ± 2.7
DTN	Influent (mg/l)	1013.3 ± 46.7	985.2 ± 71.5	2736.5 ± 67.0	2674.6 ± 59.7
	Effluent (mg/L)	477.9 ± 18.8	489.6 ± 38.3	1748.4 ± 44.4	1792.9 ± 87.9
	Removal ratio (%)	52.8 ± 1.9	50.3 ± 3.9	36.1 ± 1.6	33.0 ± 3.3
DTP	Influent (mg/l)	289.2 ± 13.9	264.7 ± 17.5	383.1 ± 7.4	378.7 ± 6.3
	Effluent (mg/l)	116.3 ± 10.4	131.5 ± 16.4	195.4 ± 10.9	228.4 ± 20.6
	Removal ratio (%)	59.8 ± 3.6	50.3 ± 6.2	49.0 ± 2.8	39.7 ± 5.4
pH	Influent (−)	4.08 ± 0.01	3.98 ± 0.01	4.07 ± 0.03	4.18 ± 0.00
	Effluent (−)	7.28 ± 0.27	6.17 ± 0.65	8.00 ± 0.15	8.49 ± 0.07
OD_660_	Effluent (−)	32.7 ± 2.1	31.4 ± 3.4	34.9 ± 1.2	36.2 ±1.5

^a^The results of barely *shochu* wastewater (diluted twice) previously reported (12) were shown for reference.

Data are means ± standard deviation of samples of each cycle.

### Effect of pH control on barley *shochu* wastewater treatment

When SSW and BSW were treated by *C. utilis* UNA1, the pH increased from about 4.0 to 7.28 – 8.0 (Table 2). The preferred pH of *C. utilis* cultures is around 4.5 to 6.0. Previously it was shown that the consumption of organic acids by fungi and yeasts caused the pH to change from mildly acidic to mildly alkaline (Tsuyumu et al. 2003; Watanabe et al. 2009). We also thought that the remaining cations (K^+^ and Ca^2+^) and ammonium ion generation by deaminization would raise the pH to mildly alkaline. To improve the treatment efficiency and cell growth, we investigated the effect of lowering the pH to the value preferred by *C. utilis* by acid addition.

**Figure 1:**
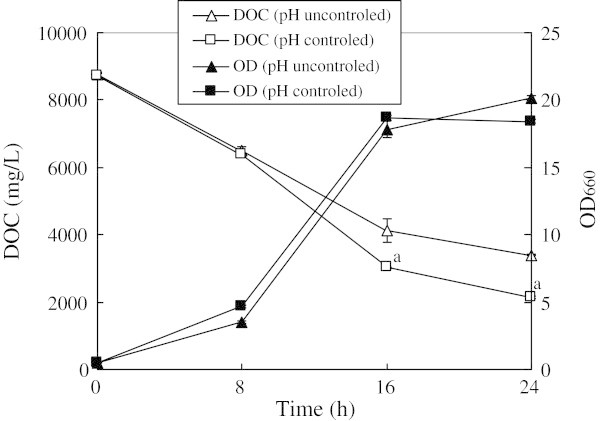
**Effect of pH control on cell growth and DOC removal treating 5-times diluted barley shochu wastewater.** The time course of the DOC concentration of pH uncontrolled (open triangle) and pH controlled (open square), and the cell growth (OD_660_) of pH uncontrolled (closed triangle) and pH controlled (closed square). The results were shown as average of three different experiments. The error bars shows standard divisions a Significantly different from pH uncontrolled (*p* < 0.05) by *t*-test.

During 8 to 16 h cultivation with 5-times diluted BSW, maintaining the pH at 5.0 with H_2_SO_4_ raised the cell growth (OD_660_) compared to no pH control (Figure 1), and increased the removal of DOC (Figure 1). On the other hand, after 24 h cultivation, cell growth was lower with pH control than without it (Figure 1). Unexpectedly, the amounts of DTN and DTP that were removed were not affected by pH control (Table 3). However, the increased removal of DOC by pH control indicates that process performance can be kept high when more concentrated SW is treated.

**Table 3:** **The effect of pH control on process performance of batch cultivation with** ***C. utilis*** **UNA1 using 5-times diluted barley** ***shochu*** **wastewater**

	pH uncontrolled	pH controlled
OD_660_ (−)	20.1 ± 0.2	18.4 ± 0.1
pH (−)	7.58 ± 0.53	5.00 ± 0.05
DOC removal amount (mg/l)	5337 ± 4	6597 ± 49
DOC removal ratio (%)	61.1 ± 0.0	75.5^a^ ± 0.6
DTN removal amount (mg/l)	596.5 ± 2.9	563.0 ± 3.5
DTN removal ratio (%)	44.2 ± 0.2	41.4 ± 0.3
DTP removal amount (mg/l)	137.9 ± 1.3	142.9 ± 0.9
DTP removal ratio (%)	75.1 ± 0.7	75.8 ± 0.5

Data are means ± standard deviation of three independent experiments.

^a^Significantly different from pH uncontrolled (*p* < 0.05) by *t*-test.

### Effect of fed-batch cultivation on *shochu* wastewater treatment

*C. utilis* UNA1 (and *C. utilis* IFO1086) cannot grow in non-diluted BSW in batch cultivation, and thus dilution with water by 4 – 5 times is needed. However, dilution of BSW requires that the treatment tank be larger, which increases the operational costs. On the other hand, the culture could be kept stable and efficiently treated by repeated-batch cultivation by replacing half the volume of treated wastewater with new 2-times-diluted BSW (Watanabe et al. 2013a). Therefore, we investigated the effect of continuous feeding into large amounts of culture on SW treatment by *C. utilis* UNA1.

### Treatment of barley *shochu* wastewater

By using feeding cultivation and maintaining the pH at 5.0 with H_2_SO_4_, 62.3% of DOC was stably removed from non-diluted BSW (Table 4). This value was higher than that of repeated-batch cultivation with two-times diluted BSW (Table 2). Furthermore, the cell growth (OD_660_) of feeding cultivation with non-diluted BSW (79.1) was twice that of repeated-batch cultivation with two-times diluted BSW (Tables 2, 4). About 16.7 kg of freeze-dried yeast biomass was obtained from 1 ton of non-diluted BSW cultivation.

**Table 4:** **Summary performances of fed-batch cultivation by** ***C. utilis*** **UNA1**

		Barley	Mixed
DOC	Influent (mg/l)	41969 ± 91	31598 ± 359
	Effluent (mg/l)	15573 ± 1315	10830 ± 617
	Removal ratio (%)	62.9 ± 3.1	65.7 ± 2.0
DTN	Influent (mg/l)	6113.8 ± 83.5	3660.1 ± 84.8
	Effluent (mg/l)	3764.7 ± 224.7	1894.1 ± 19.7
	Removal ratio (%)	38.4 ± 3.7	48.3 ± 0.5
DTP	Influent (mg/l)	787.0 ± 6.2	542.0 ± 2.2
	Effluent (mg/l)	436.7 ± 34.7	229.2 ± 2.6
	Removal ratio (%)	44.5 ± 4.4	57.7 ± 0.5
OD_660_	Effluent (−)	79.1 ± 1.5	68.4 ± 0.7

pH value was controlled at 5.0 with H_2_SO_4_.

Data are means ± standard deviation of three independent experiments.

### Treatment of mixed *shochu* wastewater

In order to concentrate SSW and to dilute BSW, we treated a 1:1 mixture of SSW and BSW (MSW). With repeated-batch cultivation, cell growth in MSW was about twice that in non-diluted SSW (Tables 2, 4). About 14.4 kg freeze-dried yeast biomass was obtained from 1 ton of MSW cultivation. Furthermore, removal ratios of DOC, DTN, and DTP of MSW treatment were higher than those of BSW (Table 4). Therefore, these results indicate that feeding cultivation of MSW with controlled pH is useful for both wastewater treatment and cell biomass production.

A marine thraustochytrid, *Schizochytrium* sp. strain KH105, was used to produce polyunsaturated fatty acids and xanthophylls from SW (v2006). However, it did not utilize and remove organic compounds from SW, and thus glucose addition was needed. In contrast, *C. utilis* UNA1 can utilize and removed over 60% of DOC, resulting in a large amount of yeast sludge biomass (Table 4).

Regarding wastewater treatment, over 30% of DOC, DTN, and DTP were still remained in yeast treated SW (Table 4). We previously confirmed that effluent of treated BSW was efficiently treated by a combination of nitrification/denitrification cycle treatment and activated sludge process (Watanabe et al. 2013a; Watanabe et al. 2009). In a laboratory-scale demonstration, 50 cycles (25 days) removed 98.9% of DOC, 95.7% of DTN, and 94.1% of DTP from BSW (Watanabe et al. 2013a). Thus, the remaining DOC, DTN, and DTP of yeast treated SW could be easily removed by conventional treatment methods.

### Chemical composition of *C. utilis* in each treatment process

The chemical compositions of yeast sludge biomass generated in each treatment process, including protein, fat, fiber, soluble non-nitrogen, ash, and minerals are shown in Table 5. With repeated-batch treatment, the crude protein contents of yeast sludge biomass generated in BSW (58.5% and 59.9%) were higher than those in SSW (35.6% and 36.0%). On the other hand, with fed-batch cultivation, the chemical compositions of *C. utilis* UNA1 differed little between BSW and MSW. Therefore, this indicates that MSW is useful for stabilizing yeast components. Using pH control with fed-batch cultivation had little effect on the total protein contents of *C. utilis* UNA1 generated in BSW.

**Table 5:** **General compositions of yeast sludge biomass generated in each treatment process with** ***shochu*** **wastewater**

Composition	Repeated-batch cultivation				Fed-batch cultivation^a^	
(% of dry matter)	Barley^b^	Barley^b^	Sweet-potato	Sweet-potato	Barley	Mixed
	IFO1086	UNA1	IFO1086	UNA1	UNA1	UNA1
Crude protein	58.5^c^	59.9^c^	35.6^c^	36.0^c^	58.2	56.9
Crude fat	1.0	1.2	0.2	0.2	1.0	0.2
Crude fiber	2.3	3.7	7.2	6.8	2.1	4.3
Soluble non-nitrogen	28.7	25.4	42.9	42.6	28.8	27.9
Crude ash	9.5	9.8	14.1	14.4	9.9	10.7
Ca	0.15	0.17	0.08	0.10	0.07	0.09
P	1.67	1.60	1.69	1.70	1.58	1.55
Mg	0.25	0.22	0.25	0.25	0.15	0.16
K	1.22	1.28	2.64	2.67	1.28	1.67

These experiments were conducted in triplicate.

^a^pH value was controlled at 5.0 with H_2_SO_4_.

^b^The results of barely *shochu* wastewater (diluted twice) treated with repeated-batch cultivation previously reported (12) were shown for reference.

^c^Significantly different barley to sweet potato (*p* < 0.05) by *t*-test.

The amino acid compositions of yeast sludge biomass made with the two types of SW were similar (Table 6). Yeast sludge biomass protein contains considerable amounts of essential amino acids. The lysine and proline contents of yeast sludge biomass generated in BSW treatment process were lower and higher than those in SSW, respectively. Using H_2_SO_4_ to control pH and fed-batch cultivation increased methionine content of *C. utilis* UNA1. Because methionine contains sulfur (S), the H_2_SO_4_ used to control pH would be expected to increase the synthesis of methionine by *C. utilis* UNA1. These results indicate that *C. utilis* UNA1 sludge biomass generated in SW treatment process could be a rich source of yeast extract and animal feed protein.

**Table 6:** **Amino acid compositions of yeast sludge biomass generated in each treatment process with** ***shochu*** **wastewater**

Amino acid	Repeated-batch cultivation				Fed-batch cultivation^a^	
(% of crude protein)	Barley	Barley	Sweet-potato	Sweet-potato	Barley	Mixed
	IFO1086	UNA1	IFO1086	UNA1	UNA1	UNA1
Arginine^b^	4.47	4.32	4.58	4.06	4.76	5.19
Glycine	4.32	4.29	4.76	4.41	4.13	4.53
Histidine^b^	2.49	2.12	2.02	1.73	2.48	2.23
Isoleucine^b^	3.91	3.93	4.11	4.08	3.87	4.10
Leucine	3.87	4.07	5.29	5.84	3.96	4.64
Lysine^b^	0.56	0.89	3.73	5.21	0.76	2.21
Methionine^b^	0.97	0.77	1.55	1.37	2.27^c^	1.99^c^
Phenylalanine^b^	8.21	7.64	6.41	4.96	7.83	6.99
Tyrosine	4.73	4.21	2.37	1.96	4.46	4.01
Valine^b^	3.87	3.91	4.90	4.93	4.00	4.53
Serine	3.97	4.01	5.14	5.17	4.17	4.84
Alanine	1.87	2.34	2.99	3.73	2.06	2.47
Aspartic acid	2.02	2.52	3.47	4.5	2.15	2.87
Glutamic acid	3.98	4.66	5.83	7.09	4.17	4.95
Proline	13.04	12.74	5.60	3.92	12.50	7.40
Threonine^b^	5.12	5.05	5.76	5.54	4.93	5.54

These experiments were conducted in triplicate.

^a^pH value was controlled at 5.0 with H_2_SO_4_.

^b^Essential amino acids.

^c^Significantly different from repeated-batch cultivation (*p* < 0.05) by *t*-test.

## Conclusions

In the present study, we investigated the cultivation process of highly concentrated SW in order to effectively treat wastewater and to produce large amounts of yeast sludge biomass to reduce operational costs. With fed-batch cultivation using a 5-L jar fermenter, controlling pH value at 5.0 with H_2_SO_4_, 62.9% of DOC, 38.4% of DTN, and 44.5% of DTP were stably removed from non-diluted BSW, and about 16.7 kg of freeze-dry yeast biomass was obtained. Furthermore, mixed with relatively thin SSW 1:1, removal ratios of DOC, DTN, and DTP were increased, and 14.4 kg of freeze-dry yeast biomass was obtained. Yeast sludge biomass generated in BSW and MSW contains about 60% of crude protein. Thus, they are useful for yeast extract source and animal feed protein. Furthermore, using H_2_SO_4_ to control pH supplied the wastewater with sulfur (S) appeared to raise the methionine content of yeast sludge biomass.

*Shohu* is a Japanese traditional liquor and its method of production is similar to the methods of whisky and brandy. Whisky distillery wastewater can be treated with yeast (Yamamoto et al. 1986). Thus, yeast should have the same advantages for alcohol distillery wastewater as it has for SW.
